# Performance of rotation forest ensemble classifier and feature extractor in predicting protein interactions using amino acid sequences

**DOI:** 10.1186/s12864-019-6304-y

**Published:** 2019-12-24

**Authors:** Alhadi Bustamam, Mohamad I. S. Musti, Susilo Hartomo, Shirley Aprilia, Patuan P. Tampubolon, Dian Lestari

**Affiliations:** Department of Mathematics, Faculty of Mathematics and Natural Science, Universitas Indonesia, Depok, 16424 Indonesia

**Keywords:** Amino acid sequences, Global encoding, Human immunodeficiency virus type 1, Protein interaction prediction, Pseudo-substitution matrix representation, Rotation forest

## Abstract

**Background:**

There are two significant problems associated with predicting protein-protein interactions using the sequences of amino acids. The first problem is representing each sequence as a feature vector, and the second is designing a model that can identify the protein interactions. Thus, effective feature extraction methods can lead to improved model performance. In this study, we used two types of feature extraction methods—global encoding and pseudo-substitution matrix representation (PseudoSMR)—to represent the sequences of amino acids in human proteins and Human Immunodeficiency Virus type 1 (HIV-1) to address the classification problem of predicting protein-protein interactions. We also compared principal component analysis (PCA) with independent principal component analysis (IPCA) as methods for transforming Rotation Forest.

**Results:**

The results show that using global encoding and PseudoSMR as a feature extraction method successfully represents the amino acid sequence for the Rotation Forest classifier with PCA or with IPCA. This can be seen from the comparison of the results of evaluation metrics, which were >73*%* across the six different parameters. The accuracy of both methods was >74*%*. The results for the other model performance criteria, such as sensitivity, specificity, precision, and F1-score, were all >73*%*. The data used in this study can be accessed using the following link: https://www.dsc.ui.ac.id/research/amino-acid-pred/.

**Conclusions:**

Both global encoding and PseudoSMR can successfully represent the sequences of amino acids. Rotation Forest (PCA) performed better than Rotation Forest (IPCA) in terms of predicting protein-protein interactions between HIV-1 and human proteins. Both the Rotation Forest (PCA) classifier and the Rotation Forest IPCA classifier performed better than other classifiers, such as Gradient Boosting, K-Nearest Neighbor, Logistic Regression, Random Forest, and Support Vector Machine (SVM). Rotation Forest (PCA) and Rotation Forest (IPCA) have accuracy, sensitivity, specificity, precision, and F1-score values >70*%* while the other classifiers have values <70*%*.

## Background

Proteins are polymers that are composed of amino acid monomers associated with peptide bonds, and they are essential for the survival of an organism. According to [1], a protein is a linear, chain-like polymer molecule comprising 10 to thousands of monomer units that are connected like beads in a necklace, with each monomer, in turn, comprising 20 natural amino acids. Proteins play an important role in forming the structural components of organisms, and they can also carry out the metabolic reactions needed to sustain life [2]. As essential macromolecules, proteins rarely act as isolated agents; instead, they must interact with other proteins to perform their functions properly [3]. Protein interactions play a central role in the many cellular functions carried out by all organisms. Thus, when irregularities occur in protein interactions, bodily malfunctions, such as autoimmune conditions, cancer, or even virus-borne diseases, can arise.

Widespread recognition of the participation of proteins in all organismal cellular processes has guided researchers to predict protein function through the sequencing of amino acids or protein structures on the basis of their interactions. Because most protein functions are driven by interactions with other proteins, developing a better understanding of protein structures should lead to a clearer picture of the impact and benefits of protein interactions [4]. Protein interactions also play a central role in medical research, as it is often necessary to understand them when developing disease-curing drugs designed to prevent or break the interactions between proteins that can result in disease.

The study of protein interactions generally involves the use of either experimental or computational methods. Experimental methods, such as *Yeast Two-Hybrid* (Y2H), *Tandem Affinity Purification*, and *Mass Spectrometric Protein Complex Identification* (MS-PCI), are known to have a number of disadvantages, including substantial time requirements for identifying protein interactions and the ability to identify only a small part of the overall protein interaction, which can potentially lead to significant mistakes in terms of research outcomes [5]. Usually, a graph can represent protein-protein interactions (PPIs). The nodes represent the protein, and the edges represent the interactions between the proteins [6]. However, the graph representation can only make clusters of interaction. To predict new interactions, we have to use the amino acid sequencing.

When identifying protein-protein interactions using amino acid sequencing, computational methods must solve two major problems: effectively representing a sequence as a feature vector that can be analyzed and designing a model that can identify protein interactions accurately and quickly. To solve these problems, computational methods generally apply a two-stage approach involving feature extraction followed by machine learning [7].

Effective feature extraction methods are required to represent sequences of amino acids as whole proteins. An effective feature extraction method will provide better model performance by skillfully extracting potential information from an amino acid sequence and representing it as feature vectors for further analysis via machine learning [7]. The feature extraction method has become one of the most important benchmarks for ensuring the successful classification of proteins based on their constituent amino acids. The success, or even failure, of a classification method in identifying protein interactions based on the sequence of amino acids cannot be seen only from the point of view of whether or not the classification method is effective; it must also be determined based on how well a feature extraction method represents a sequence of amino acids in the input feature vectors to be analyzed later in the classification method. Many studies have focused on developing methods for the feature extraction of amino acid sequences for use in further machine learning analysis. Sharma et al. [8] used feature extraction techniques to recognize protein folds that use the bi-gram feature by using position-specific scoring matrix (PSSM) and Support Vector Machine (SVM) as the classifiers. Dehzangi et al. [9] used the bi-gram feature technique for predicting protein subcellular localization for *Prokaryotic* microorganisms, i.e., Gram-positive and Gram-negative bacteria. Huang et al. [7] developed a successful feature extraction approach called global encoding, which has come to play an important role in weighted sparse representation modeling as a classifier for predicting protein interactions from their amino acid sequences. In a related study, pseudo-substitution matrix representation (PseudoSMR) features were also found to be useful in applying the weighted sparse representation method to the identification of interactions between proteins [3].

Machine learning methods adopt algorithms or mathematical models to perform classification, and they have been used to develop multiple classifier systems (MCSs). Machine learning can be implemented either by applying multiple classification methods to a given dataset or by applying a single method to several different data subsets. Most researchers have used the following classifiers: Gradient Boosting, K-Nearest Neighbor, Logistics Regression, Random Forest, and SVM. For example, SVM and Naïve Bayes classifier has been used for analyzing the texture of the brain 3D MRI images [10]. In 2006, Rodriguez et al. [11] proposed Rotation Forest as an ensemble classifier method, a type of MCS that uses compound decision trees to perform classification on several data subsets. This method involves the application of bagging and Random Forest algorithms to perform principal component analysis (PCA), and then matrix rotation on the datasets, which are compiled into compound decision trees. The rotation process produces decision trees that are mutually independent. Although the PCA is applied, all principal components (PCs) are still used to build the decision trees to ensure the completeness of the data. This method has been shown to perform well as a classification method for identifying protein interactions based on amino acid sequences [5, 12].

The success of feature extraction methods, such as global encoding and PseudoSMR, in extracting the features of amino acid sequences for use as input data, together with the usefulness of the Rotation Forest method as a classification method for predicting amino acid sequences, suggests that these methods could be combined into a system to successfully predict PPIs, which was the goal of this study. We also assessed the performance of the Rotation Forest classifier under two different transformation methods: PCA and independent principal component analysis (IPCA). Yao et al. introduced IPCA as a method for successfully combining the respective advantages of PCA and independent component analysis (ICA) for uncovering independent principal components (IPCs) [13].

Kuncheva and Rodriguez [14] demonstrated that PCA could be successfully applied as a Rotation Forest transformation method, and that it was more accurate than random projection and nonparametric discriminant analysis. The higher accuracy of PCA is due to its ability to produce rotational matrices with very small correlations, characterized by a reduced cumulative proportion of matrix diversity, which enables the formation of mutually independent decision trees within an ensemble system. Thus, PCA guarantees a diversity of decision trees under the Rotation Forest method in the same manner as the separation of random data free variables. This prevents the production of large numbers of allegations that can cause the model to experience inconsistencies in decision-making. Therefore, PCA can play an important role in improving the accuracy of the Rotation Forest method while ensuring the diversity of the established ensemble systems.

As mentioned earlier, Yao et al. [13] developed a dimensional reduction method that works in a manner similar to PCA. Their method transforms an initial data group to reduce its dimensionality while maintaining a transformed component that can represent the data as a whole. The method applies PCA in an initial stage to produce a loading matrix, which contains the coefficients of the linear combination of the initial free data variables used to produce the PCs, for input into an ICA stage [13]. Because the PCA loading matrix for biological data will still contain a large amount of noise, ICA is used to generate a new loading matrix that contains little or no noise from which potential data can be extracted. ICA is used in this process because of its known ability to find hidden (latent) variables in noisy data [15]. The IPCA process is used to produce an independent loading vector matrix that is then applied as a rotation matrix to the initial data group to produce a set of IPCs.

The IPCA method is often used as a clustering method, and to perform dimensional reduction. In the present study, IPCA was not used to perform these tasks; instead, it was applied in the Rotation Forest method to transform initial free data variables into new variables within an independent loading vector matrix in which all of the PCs in the PCA loading matrix were retained. This use of IPCA as a method of transformation under Rotation Forest for predicting protein interactions based on amino acid sequences represents a novel approach in the literature; accordingly, it was further tested by comparing the performance of the Rotation Forest method by applying global encoding for feature extraction under both PCA and IPCA. The proposed method was then used to predict the amino acid sequence of Human Immunodeficiency Virus type 1 (HIV-1) to identify newly identified human proteins that can interact with HIV-1 proteins based on a comparison between the respective sequences in both organisms.

### HIV

Although viruses are the smallest reproductive structures, they have a substantial range of abilities. A virus generally consists of four to six genes that are capable of taking over the biological processes within a host cell during its reproductive process [16]. The virus forces the host cell to produce new viruses by inserting its genetic information, in the form of DNA and viral RNA, into the cell. This process compromises the host cell to the point that it dies when the virus reproduction process is complete.

HIV attacks the human immune system. The virus is often also referred to as an intracellular obligate retrovirus because of its ability to convert single-stranded RNA into double-helix DNA within infected cells, and then merge it with the target cell’s DNA, forcing it to replicate into new viruses [16]. The targets are cells that can express CD4 receptors, which play an important role in maintaining immune system cells, such as T-lymphocytes. In fact, damage to or destruction of even one T-lymphocyte cell can lead to the failure of the entire specific immune response to attacks from harmful pathogens, even, ironically, from HIV itself [16].

HIV infects the human body through protein interactions. The HIV-linked glycoprotein 120 binds to specific T-cell receptors to produce bonding between a virus and the target cell. This bond is then reinforced by the second coordinator, which consists of a number of transmembrane receptors, such as CC Chemokine Receptor 5 (CCR5) or CXC Chemokine Receptor 4 (CXCR4) that bind through 100 interactions between the viral proteins and the target cells. Once binding has occurred, HIV glycoprotein 41 allows the virus to enter the target cell membrane, and its reverse transcriptase enzyme converts a single strand of RNA into a double-helix DNA virus that will be carried into the target cell nucleus and inserted into the cell’s DNA via an integrase enzyme. Once this occurs, the host cell becomes a provirus.

The connected DNA of the viral and human cells is transcribed by a polymerase enzyme to produce genomic RNA and mRNA. The RNA is ejected from the cell nucleus, and the mRNA undergoes a process of transition into a polypeptide, which is then incorporated with the RNA into a new viral core, and assembled on the surface of the target cell. Protease enzymes then break down the polypeptide into new proteins and other functional enzymes. This process results in new HIV viruses that are ready to infect other target cells that express the CD4 receptor. The reproduction of the HIV virus slowly creates a failure in the immune system that results in the body’s inability to fight various types of diseases and infections in a process known as opportunistic disease spread; ultimately, this can result in full-blown Acquired Immunodeficiency Syndrome.

## Results

In this study, we used *R*=2,3,4,5,6,7, and 7 for Global Encoding and *Lg*=2,3,5,6,8, and 10 for PseudoSMR. The difference in the value between *R* and *Lg* is because we wanted to compare dimensions that are not too different, which can be caused by differences in the values of those two parameters. We also used *K*=1,5,10,15,20, and *p*/3 and *L*=10,20,30,40,50,60,70,80,90 and 100 as the parameters in the Rotation Forest (PCA) and Rotation Forest (IPCA) methods. Tables 1 and 2 show the performance evaluation results obtained from Rotation Forest (PCA) and Rotation Forest (IPCA), respectively, for various values of *L* and *K*, as well as the *R* parameters, and with global encoding combined with both methods. For both methods, the best scores tended to occur for *K*=*p*/3 at various values of *L* and *R*. The results presented in both tables indicate that using global encoding as a feature extraction method successfully represents sequences of amino acids; this is seen from a comparison of the evaluation metric results, which was >73*%* across the six distinct parameters used in global encoding.

**Table 1:** Performance of Rotation Forest (PCA) combined with global encoding

R	Dim.	Acc.	Sen.	Spe.	Pre.	F1-s.
2	15,350 ×120	77.85	78.10	77.59	78.50	78.29
3	15,350 ×180	78.26	78.56	77.95	78.80	78.63
4	15,350 ×240	79.50	79.91	79.07	79.78	79.33
5	15,350 ×300	78.57	78.93	78.18	78.97	78.75
6	15,350 ×360	78.96	79.59	78.30	78.88	79.27
7	15,350 ×420	78.98	79.01	78.50	79.27	79.18

**Table 2:** Performance of Rotation Forest (IPCA) combined with global encoding

R	Dim.	Acc.	Sen.	Spe.	Pre.	F1-s.
2	15,350 ×120	74.03	73.74	74.35	76.07	74.71
3	15,350 ×180	75.09	74.91	75.29	76.79	75.47
4	15,350 ×240	76.00	76.04	75.96	77.17	76.53
5	15,350 ×300	75.79	75.49	76.12	77.64	76.18
6	15,350 ×360	76.79	76.48	77.11	78.54	77.50
7	15,350 ×420	77.19	76.65	77.81	79.39	77.99

It is further seen that the accuracy of both methods is >74*%*, indicating that both correctly predict interactions between HIV-1 and human proteins in more than approximately three out of four cases. The other model performance criteria results are fairly similar to the accuracy results; all the sensitivity, specificity, precision, and F-1 score results were >73*%*. This indicates that both methods can recognize positive and negative observations >73*%* of the time with a precision >75*%*. The high degree of balance among the results reveals the high predictive capabilities of both methods [17].

A comparison of the data presented in Tables 1 and 2 reveals that the Rotation Forest (PCA) method performed better than the Rotation Forest (IPCA) method across various dimensions of global encoding. Table 1 shows that the highest accuracy obtained by the Rotation Forest (PCA) method (79.50%) occurs on the global encoding dataset with *R*=4, corresponding to a data dimensionality of 15,350×240; the highest accuracy obtained by the Rotation Forest (IPCA) method (77.19%) occurs at *R*=7, or at a dimensionality of 15,350×420. However, changing the parameter difference (*R*) in the global encoding does not significantly affect the performance of either method, as the accuracy (Acc.), sensitivity (Sen.), specificity (Spe.), precision (Pre.), and F1-score (F1-s.) values all lie within a range of two percentage points. This suggests that it is possible to successfully represent amino acid sequences using smaller dimensionalities (i.e., lower values of *R*) in the global encoding. Conversely, increasing the number of global encoding parameters will increase the dimensionality of the data, which, in turn, will increase the time complexity and memory requirements of an algorithm used to solve a problem.

The data presented in Tables 3 and 4 show the performance results obtained, respectively, by the Rotation Forest (PCA) and Rotation Forest (IPCA) methods using the PseudoSMR dataset. The former performs best at *Lg*=5, whereas the latter performs best at *Lg*=8. However, the respective performance evaluation criteria results differ within a limited range of 0.02 to 0.03, indicating that both methods have good predictive ability. This result also confirms that increasing the *Lg* parameter used in the PseudoSMR feature method does not result in a significant difference in model performance, suggesting that a small *Lg* parameter can successfully represent amino acid sequences. As with the *R* global encoding pattern, the size of the *Lg* parameter in the PseudoSMR feature should be considered because any increases in it will increase the dimensionality of the data and, thus, the computational complexity.

**Table 3:** Performance of Rotation Forest (PCA) combined with PseudoSMR

Lg	Dim.	Acc.	Sen.	Spe.	Pre.	F1-s.
2	15,350 ×120	77.78	77.48	79.37	78.59	79.44
3	15,350 ×180	79.41	79.63	80.19	79.40	79.44
5	15,350 ×240	80.24	81.22	79.36	79.28	80.21
6	15,350 ×300	78.29	79.19	80.55	78.44	78.95
8	15,350 ×360	79.37	80.94	80.80	80.61	79.74
10	15,350 ×420	78.89	79.57	80.41	78.63	79.09

**Table 4:** Performance of Rotation Forest (IPCA) combined with PseudoSMR

Lg	Dim.	Acc.	Sen.	Spe.	Pre.	F1-s.
2	15,350 ×120	76.27	76.32	77.98	76.89	76.94
3	15,350 ×180	75.94	76.97	78.98	76.97	76.97
5	15,350 ×240	77.48	77.83	78.17	77.83	77.83
6	15,350 ×300	76.89	79.19	79.31	78.44	77.53
8	15,350 ×360	77.83	76.87	79.92	79.27	78.04
10	15,350 ×420	76.74	76.46	79.83	78.59	77.33

From the results listed in Tables 1, 2, 3 and 4, it is seen that Rotation Forest (PCA) outperforms Rotation Forest (IPCA) on both the global encoding and PseudoSMR datasets. It is also seen that both feature extraction methods are skillful at representing sequences of amino acids as vector inputs for further analysis, even when small *R* or *Lg* parameters are used. *K* and *L* are the most important parameters for determining the performance of Rotation Forest under the grid search method. In the assessments above, we set *K*=*p*/3 and *L*=90 as these values tended to result in strong performance by both the PCA and the IPCA model variants.

From the results presented in Tables 1, 2, 3 and 4, it is seen that the Rotation Forest (PCA) method outperforms the Rotation Forest (IPCA) method on both the global encoding and PseudoSMR datasets. It is also seen that both feature extraction methods effectively represent sequences of amino acids as vector inputs for further analysis, even when small *R* or *Lg* parameters are used. *K* and *L* are the most important parameters for determining the performance of the Rotation Forest classifier under the grid search method. In the assessments above, we set *K*=*p*/3 and *L*=90 because these values tended to result in strong performance by both the PCA and the IPCA model variants.

From the results listed in Tables 5, 6, 7, 8, 9, 10, 11, 12, 13 and 14, it can be seen that classifiers, such as Gradient Boosting, K-Nearest Neighbor, Logistic Regression, Random Forest, and SVM, cannot surpass the success of Rotation Forest (PCA), which outperforms Rotation Forest (IPCA) in terms of accuracy, sensitivity, specificity, and precision.

**Table 5:** Performance of Gradient Boosting combined with global encoding

R	Dim.	Acc.	Sen.	Spe.	Pre.	F1-s.
2	15,350 ×120	67.17	69.83	64.45	66.73	68.25
3	15,350 ×180	67.87	70.50	65.19	67.41	68.92
4	15,350 ×240	67.77	69.78	65.72	67.51	68.63
5	15,350 ×300	67.64	70.14	65.09	67.23	68.65
6	15,350 ×360	67.90	68.85	66.93	68.01	68.43
7	15,350 ×420	68.00	69.16	66.82	68.04	68.59

**Table 6:** Performance of K-Nearest Neighbor combined with global encoding

R	Dim.	Acc.	Sen.	Spe.	Pre.	F1-s.
2	15,350 ×120	61.13	64.72	57.45	60.83	62.72
3	15,350 ×180	61.52	64.78	58.19	61.27	62.97
4	15,350 ×240	60.89	64.16	57.56	60.68	62.37
5	15,350 ×300	60.84	63.90	57.71	60.68	62.25
6	15,350 ×360	61.59	64.41	58.72	61.44	62.89
7	15,350 ×420	61.88	64.88	58.82	61.67	63.23

**Table 7:** Performance of Logistic Regression combined with global encoding

R	Dim.	Acc.	Sen.	Spe.	Pre.	F1-s.
2	15,350 ×120	58.18	57.76	58.61	58.76	58.26
3	15,350 ×180	58.81	59.52	58.08	59.18	59.35
4	15,350 ×240	58.39	58.02	58.77	58.96	58.49
5	15,350 ×300	59.22	59.36	59.08	59.70	59.53
6	15,350 ×360	60.16	60.03	60.29	60.69	60.36
7	15,350 ×420	60.53	60.91	60.14	60.94	60.92

**Table 8:** Performance of Random Forest combined with global encoding

R	Dim.	Acc.	Sen.	Spe.	Pre.	F1-s.
2	15,350 ×120	75.20	71.02	79.46	77.93	74.31
3	15,350 ×180	75.17	71.43	78.99	77.63	74.40
4	15,350 ×240	75.01	71.02	79.09	77.62	74.17
5	15,350 ×300	75.46	71.27	79.73	78.21	74.58
6	15,350 ×360	75.66	70.55	80.88	79.03	74.55
7	15,350 ×420	76.84	72.72	81.04	79.66	76.03

**Table 9:** Performance of Support Vector Machine combined with global encoding

R	Dim.	Acc.	Sen.	Spe.	Pre.	F1-s.
2	15,350 ×120	60.84	95.20	25.75	56.70	71.07
3	15,350 ×180	61.62	95.31	27.22	57.21	71.50
4	15,350 ×240	61.62	94.79	27.75	57.26	71.39
5	15,350 ×300	61.57	94.07	28.38	57.29	71.21
6	15,350 ×360	62.01	94.33	29.02	57.57	71.50
7	15,350 ×420	61.91	93.91	29.23	57.54	71.36

**Table 10:** Performance of Gradient Boosting combined with PseudoSMR

Lg	Dim.	Acc.	Sen.	Spe.	Pre.	F1-s.
2	15,350 ×120	67.56	70.01	65.05	67.28	68.62
3	15,350 ×180	67.77	70.88	64.57	67.25	69.02
5	15,350 ×240	69.75	72.46	66.98	69.14	70.76
6	15,350 ×300	68.71	70.94	66.42	68.44	69.66
8	15,350 ×360	68.99	72.07	65.84	68.41	70.19
10	15,350 ×420	68.92	70.88	66.90	68.73	69.79

**Table 11:** Performance of K-Nearest Neighbors combined with PseudoSMR

Lg	Dim.	Acc.	Sen.	Spe.	Pre.	F1-s.
2	15,350 ×120	66.36	70.42	62.20	65.66	67.96
3	15,350 ×180	66.86	70.68	62.94	66.18	68.36
5	15,350 ×240	66.13	69.88	62.30	65.43	67.58
6	15,350 ×300	65.95	70.22	61.56	65.22	67.62
8	15,350 ×360	66.21	70.58	61.72	65.43	67.90
10	15,350 ×420	66.31	70.27	62.25	65.64	67.88

**Table 12:** Performance of Logistics Regression combined with PseudoSMR

Lg	Dim.	Acc.	Sen.	Spe.	Pre.	F1-s.
2	15,350 ×120	62.56	64.51	60.56	62.67	63.57
3	15,350 ×180	61.70	64.35	58.98	61.69	62.99
5	15,350 ×240	63.11	65.86	60.29	62.88	64.33
6	15,350 ×300	63.16	64.51	61.77	63.40	63.95
8	15,350 ×360	63.47	64.92	61.99	63.67	64.29
10	15,350 ×420	63.37	64.56	62.14	63.64	64.10

**Table 13:** Performance of Random Forest combined with PseudoSMR

Lg	Dim.	Acc.	Sen.	Spe.	Pre.	F1-s.
2	15,350 ×120	75.51	71.66	79.46	78.17	74.77
3	15,350 ×180	75.12	70.37	79.99	78.31	74.13
5	15,350 ×240	76.89	72.61	81.25	79.82	76.05
6	15,350 ×300	76.06	71.86	80.36	78.97	75.25
8	15,350 ×360	76.92	72.63	81.31	79.95	76.12
10	15,350 ×420	75.72	71.35	80.20	78.72	74.85

**Table 14:** Performance of Support Vector Machine combined with PseudoSMR

Lg	Dim.	Acc.	Sen.	Spe.	Pre.	F1-s.
2	15,350 ×120	63.26	68.11	58.29	62.63	65.25
3	15,350 ×180	62.38	67.18	57.44	61.84	64.40
5	15,350 ×240	65.27	71.17	59.24	64.07	67.43
6	15,350 ×300	64.62	70.16	58.92	63.68	66.76
8	15,350 ×360	65.55	71.45	59.50	64.42	67.76
10	15,350 ×420	65.11	70.78	59.29	64.09	67.27

### Sensitivity analysis of *K* and *L* rotation forest parameters

**Fig. 1:**
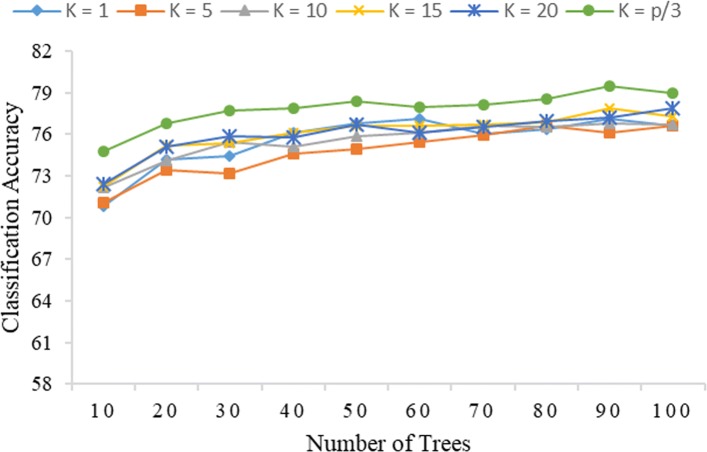
Correlation of the level of accuracy of classification of Rotation Forest (PCA) with *K* and *L* under global encoding (*R*=4)

**Fig. 2:**
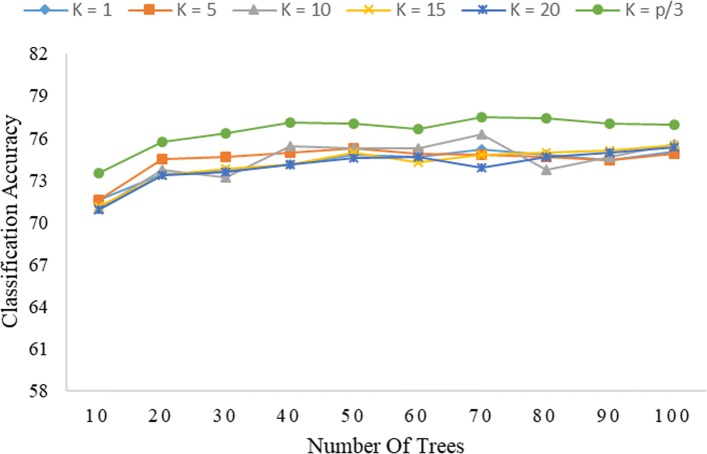
Correlation of the level of accuracy of classification of Rotation Forest (PCA) with *K* and *L* under PseudoSMR (*Lg*=5)

Figures 1 and 2 show that, at a given value of *K*, the classification accuracy of the Rotation Forest (PCA) method tends to increase with the value of *L* under both global encoding and PseudoSMR. The accuracy of classification is seen to be maximum at *K*=*p*/3; this result is consistent with the finding in [11], which also reported optimal Rotation Forest accuracy at *K*=*p*/3. Thus, at *K*=*p*/3, the ability of PCA to ensure diversity in the ensemble system through its transformation process is optimized. Moreover, it appears that Rotation Forest requires only a few decision trees to obtain good performance results, as it was observed that increasing the value of *L* tends to result in converging performance. It should also be noted that increasing *L* will lead to increased computational complexity and time.

**Fig. 3:**
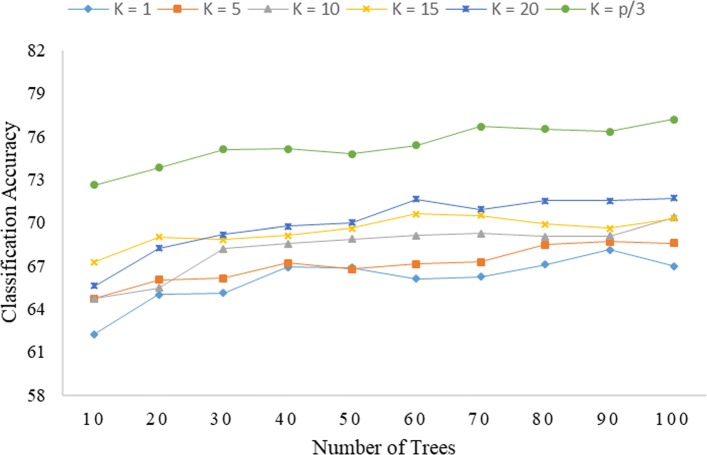
Correlation of the level of accuracy of classification of Rotation Forest (IPCA) with *K* and *L* under global encoding (*R*=7)

**Fig. 4:**
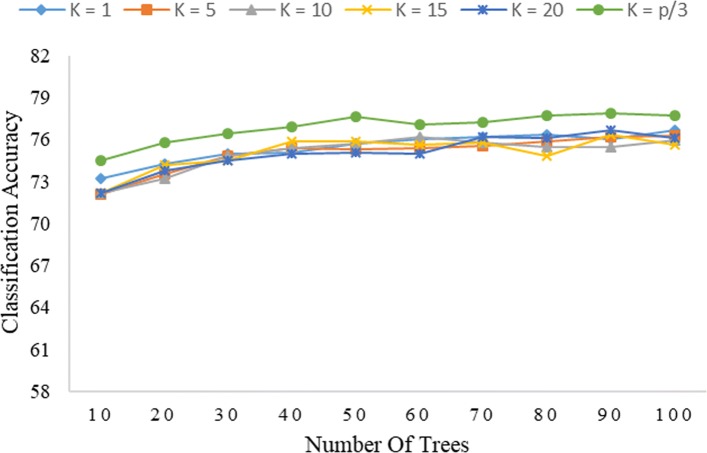
Correlation of the level of accuracy classification of Rotation Forest (IPCA) with the values of *K* and *L* combined with PseudoSMR (*Lg*=8)

As seen from Fig. 3, the global encoding dataset tends to produce Rotation Forest (IPCA) results similar to those of Rotation Forest (PCA). Furthermore, Rotation Forest (IPCA) is also most accurate at *K*=*p*/3, while, generally, producing the worst results at *K*=1. This corresponds to no separation of the original free variables, with the PCA simply turning all the free variables over to the process of forming a decision tree in each classifier. This emphasizes the importance of the feature separation process in improving the performance of Rotation Forest (IPCA) in terms of producing a diversity of combined decision trees from the global encoding dataset. As seen in Fig. 4, the PseudoSMR dataset also produces similar results for both Rotation Forest (IPCA) and Rotation Forest (PCA), with the classifier performing best at *K*=*p*/3.

## Discussion

In this assessment, all of the PC coefficients contained in the loading matrices of both methods were used. This was done following [14], which showed that the PC coefficients with the smallest diversity have the highest influence on the process of forming a composite tree on Rotation Forest (PCA). However, in Rotation Forest (IPCA), the use of IPCA as a preliminary transformation method serves to reduce the dimensionality of the data and eliminate noise from the loading matrix prior to inputting into the ICA process. This might account for the reduced performance of Rotation Forest (IPCA) relative to Rotation Forest (PCA), as the IPCA result matrix possibly retains information that is not important because it does not select features from the initial feature set. Rotation Forest (PCA) is also likely to experience constraints when using noisy data, which can occur when the feature extraction method fails to represent a protein sequence. In such cases, the PCs generated by the PCA might be unable to extract relevant information from the data and build good decision trees. Further research is required to test these hypotheses.

Rotation Forest also requires a large computation time for large datasets or large values of *K* and *L*. This situation might be mitigated by introducing parallel computational methods in subsequent research. In the present study, we also processed, but did not include, pairs of amino acid sequence data that have similarities of more than 40%. We did this to reduce noise from the data. However, the method for determining the best similarity criteria to reduce noise from the data should be further developed. Finally, additional datasets can be used to further test the performance of the respective models, while other prediction models, aside from decision tree C4.5, can be developed to solve problems using Rotation Forest (PCA) and Rotation Forest (IPCA) methods. In this research study, we compared the model with state-of-the-art from other machine learning models, such as SVM, K-Nearest Neighbor, Random Forest, and other algorithms. It is expected that this research could provide basic ideas for further research in predicting the interactions of human proteins with HIV-1 using amino acid sequence data using the Rotation Forest method.

## Conclusion

In this study, global encoding and PseudoSMR were found to be very capable of representing series of amino acids, and the combination of these representation methods with Rotation Forest (PCA) and Rotation Forest (IPCA) resulted in generally good classification performance across the range of feature extraction parameters that were examined. The lack of significant differences in model performance suggests that both feature extraction methods perform best at relatively small values of *R* and *Lg*, as increasing either would lead to issues of increased data dimensionality and, in turn, heavier computational loads. This result affirms that research related to extracting features for sequences of amino acids in proteins must look at using good input data with dimensionality that is not too high.

The Rotation Forest (PCA) method performed best in terms of predicting protein–protein interactions between HIV-1 and human proteins using global encoding, with an accuracy, sensitivity, specificity, and precision of 79.77%, 79.91%, 79.07%, and 79.77%, respectively, at *R*=4. The Rotation Forest (IPCA) method obtained corresponding values of 77.20%, 76.65%, 77.81%, and 79.40% at *R*=7. Similarly, using PseudoSMR with Rotation Forest (PCA) resulted in an accuracy, sensitivity, specificity, and precision of 80.23%, 81.25%, 79.35%, and 79.28%, respectively, at *Lg*=5. Using PseudoSMR with Rotation Forest (IPCA) resulted in corresponding values of 77.83%, 76.87%, 79.92%, and 79.26% at *Lg*=8.

Both methods achieved optimal results at *K*=*p*/3 for various values of *L*, *R*, and *Lg*. Although Rotation Forest (PCA) was somewhat better at predicting protein–protein interactions between HIV-1 and human proteins, the difference in performance between the two classifiers was insignificant. All the PC coefficients were used in the loading matrix in this study, based on the results of Kuncheva and Rodriguez [14], who found that coefficients of PCs, with even the smallest variation, can affect the process of composite tree formation in Rotation Forest (PCA). However, further research should be conducted to determine whether the use of all the major component coefficients by Rotation Forest (IPCA) is effective, as the additional feature selection processing used by this method to eliminate noise from the loading matrix might reduce its performance relative to Rotation Forest (PCA).

## Methods

### Gold standard dataset

The data used in this study consisted of the amino acid sequences of several HIV-1 proteins, some of which are interactive with human proteins, and some are not. Both datasets were obtained from https://www.ncbi.nlm.nih.gov/, which was accessed in September 2017 in several stages. A total of 15,665 pairs of HIV-1 proteins that interact with human proteins were obtained from the website, although the data required further paring-down to eliminate cases in which individual human proteins could interact with different strains of a single HIV-1 protein to reduce data repetition.

Following the selection process mentioned above, the dataset comprised 7,760 HIV-1-human protein–protein pairs. To identify individual sequences of amino acids from within the proteins, we searched for HIV-1 and human protein amino acid lines on [18], which was accessed in September 2017, and we obtained the complete amino acid sequence for each protein in the interaction dataset. The noninteracting protein dataset was obtained by downloading the entire human protein dataset along with its corresponding amino acid sequences. In total, the human protein database contains 109,671 proteins; a random sample of proteins from this database was then compared with the interaction dataset to find mismatches (i.e., proteins in the former that were not present in the latter). Based on this search, 69,129 noninteracting proteins were identified, of which 7,760 pairs were randomly selected to provide a dataset that balanced the interacting dataset. Overall, 15,520 pairs of interacting and noninteracting human and HIV-1 protein amino acid sequences were selected for the study.

The next step was to select all the datasets that had previously been obtained. Two selection criteria were used to eliminate sequences from the dataset in this step: pairs of amino acid sequences with similarities ≥40*%* and sequences with residue lengths of <50 were excluded from further use. The remaining protein dataset, comprising 15,350 interacting and noninteracting protein pairs, was then defined as the gold standard dataset, or the golden dataset. Two distinct feature extraction methods were then applied to the golden dataset to produce vectors for input into the Rotation Forest in the next stage.

### Global encoding amino acid sequence

As mentioned in the previous sections, effective feature extraction methods for representing the sequences of amino acids within proteins produce better model performance. In general, feature extraction methods are used to extract candidate amino acid sequences as feature vectors to be inputted into a machine learning method [19]. Global encoding is a feature extraction method that was developed by Huang et al. to predict protein interactions through application of a weighted sparse representation classifier to amino acid sequences [7]. The method classifies residual or amino acid codes into six initial classes based on the psychochemical nature of each residue. It then constructs 10 groups comprising two subsets, each consisting of three different classes. The next step is to transform each sequence of amino acids into 10 binary rows corresponding to its respective group; this binary sequence is referred to as a sequence of characteristics. Each sequence of characteristics is then partitioned following a specific strategy, and the number of partitions is adjusted to the size of the used parameter (*R*). Finally, the resulting sequence of characteristic partitions is extracted into a vector feature for input into a Rotation Forest classifier. The steps of the global encoding method are given in detail, below [7].

#### Step 1: transformation of amino acid protein sequence

Each amino acid is grouped into six different classes according to the psycho-chemical characteristics of each amino acid, as shown in Table 15 [20].

**Table 15:** Classification of amino acids

Aliphatic amino acids	D1 = {A,V,L,I,M,C}
Aromatic amino acids	D2 = {F,W,Y,H}
Polar amino acids	D3 = {S,T,N,Q}
Positive amino acids	D4 = {K,R}
Negative amino acids	D5 = {D,E}
Special shape	D6 = {G,P}

Based on the information presented in Table 15, 10 groups of codes can be formed by dividing the six classes into two subsets in which each subset contains three different classes. Thus, this method produces 10 groups, each containing two subsets, each of which, in turn, contains three different classes. An example of this structure is as follows: {D1, D2, D3} and {D4, D5, D6}, {D1, D2, D4} and {D3, D5, D6}, {D1, D2, D5} and {D3, D4, D6}, {D1, D2, D6} and {D3, D4, D5}, {D1, D3, D4} and {D2, D5, D6}, {D1, D3, D5} and {D2, D4,D6}, {D1, D3, D6} and {D2, D4, D5}, {D1, D4, D5} and {D2, D3, D6}, {D1, D4, D6} and {D2, D3, D5}, {D1, D5, D6} and {D2, D3, D4}. Here, there are a total of 10 groups of two bracketed subsets of classes.

A sequence of amino acids *T*=*t*_1_,*t*_2_,…,*t*_*n*_ where *t*_1_,*t*_2_,…,*t*_*n*_ is the residue or the *i*-th amino acid code of the sequence, is transformed into 10 rows of characteristics corresponding to the 10 groups. As an illustration, we show four sequences of characteristics, which are grouped into *H*_1_(*t*_*i*_) and *H*_2_(*t*_*i*_) as follows:

1 $$ H_{1}(p_{i}) = \left \{\begin{array}{ll} 1, p_{i} \in \{D_{1},D_{2},D_{3}\}& \\ & i= 1, \dots, n,\\ 0,p_{i} \in \{D_{4},D_{5},D_{6}\} \end{array}\right.  $$

2 $$ H_{2}(p_{i}) = \left \{\begin{array}{l l} 1, p_{i} \in \{D_{1},D_{2},D_{4}\}& \\ & i= 1, \dots, n,\\ 0,p_{i} \in \{D_{3},D_{5},D_{6}\} \end{array}\right.  $$

3 $$ H_{10}(p_{i}) = \left \{\begin{array}{l l} 1, p_{i} \in \{D_{1},D_{5},D_{6}\}& \\ & i= 1, \dots, n,\\ 0,p_{i} \in \{D_{2},D_{3},D_{4}\} \end{array}\right.  $$

where *H*_*u*_(*p*_*i*_) is the sequence of u-characteristics of a given amino acid sequence and *u*=1,2,…,10.

#### Step 2: partitioning the characteristic sequence

In this stage, all the characteristic sequences of length *n* are partitioned by dividing each sequence into several subsequences of varying length. A characteristic sequence *H*_*u*_=*s*_1_,*s*_2_,…,*s*_*n*_, in which *s*_1_,*s*_2_,…,*s*_*n*_ are the elements of the sequence given by values of 0 or 1, is divided into many *R* sub-characteristic sequences, where *R* is an integer. The *k*-th subsequence of *H*_*u*_, denoted by *SubsH*_*k*_, is composed of the first ⌊*kn*/*R*⌋ numbers of *H*_*u*_.

#### Step 3: feature vector extraction

In this stage, the features of the composition and transition vectors are extracted from the characteristics of the subsequences generated in the partition stage. Two descriptors are produced in this step: (1) a composition descriptor that gives the respective frequencies of “0” and “1” in each subsequence characteristic and (2) a transition descriptor that calculates the frequency of changing from 1 to 0 or from 0 to 1 in each subsequence [7].

### PseudoSMR features

As mentioned above, in order to extract features, we used SMR, a new method that was introduced in 2011 to sequence proteins that store evolutionary information [3]. The proposed PseudoSMR method forms each sequence of proteins into an initial *N*×20 matrix, where *N* is the length of a single sequence of proteins and 20 is the total number of amino acid types. It then substitutes the membership value of each pair of amino acids into the matrix. Here, a BLOSUM62 matrix, which is often used to calculate alignment between two different protein sequences, is used. The value of the BLOSUM62 matrix is based on the observed polypeptide alignments found from sampling very large datasets.

The SMR matrix is constructed as follows:

4 $$ SMR_{i} = \left[\begin{array}{cccc} V_{1,1} & V_{1,2} & \dots & V_{1,20}\\ V_{2,1} & V_{2,2} & \dots & V_{2,20}\\ \vdots & \vdots & \ddots & \vdots\\ V_{N,1} & V_{N,2} & \dots & V_{N,20}\\ \end{array}\right]  $$

,*i*=1,2,…, total number of protein pairs,

where *V*_*i*,*j*_ denotes the possible BLOSUM62 value describing the *i*-th amino acid of the protein sequence showing the mutation value of the *y*-th amino acids in the evolutionary process. When preparing the SMR matrix, the lengths of the constituent vectors can vary depending on the length of the corresponding proteins. To ensure that all the vectors have the same length, the protein sequences and amino acid compositions within the vectors are adjusted to form the final PseudoSMR matrix, as follows:

5 $$ \small PseudoSMR(n) = \left \{\begin{array}{l} \frac{1}{N}\sum_{i=1}^{N}K(i,j),\\ \indent \indent n=1,\dots,20 \\ \indent \indent j=1,\dots,20\\\\ \frac{1}{N-lg}\sum_{i=1}^{N-lg}[K(i,j)-K(i+lg,j)]^{2},\\ \indent \indent \indent j=1,\dots,20\\ \indent \indent lg=1,\dots,Max Lg\\ \indent \indent n=20+j+20\cdot(lg-1)\\ \indent \indent Max Lg=15\\ \end{array}\right. \normalsize  $$

6 $$ K(i,j) = \frac{SMR(i,j)-\frac{1}{20}\sum_{a=1}^{20}SMR(i,a)}{\sqrt{\frac{1}{20}\sum_{a=1}^{20}(SMR(i,b)-\frac{1}{20}SMR(i,a))^{2}}}  $$

### IPCA

The rapid growth of technology and increase in research resulted in a vast pool of biological data that cannot be easily processed or represented, and new methods must be developed to uncover relevant and important information and relationships contained within these data. Although several statistical methods have been developed to address these challenges, solving the big data problem using conventional statistical methods is often difficult because the number of observations is often smaller than the corresponding number of variables, and the available data contains a large amount of noise [13]. IPCA, a new method for addressing this problem [13], is an unsupervised learning method for uncovering useful patterns by reducing the dimensionality of data through a projection process that decomposes it into more informative components. Unlike ICA, IPCA does not directly reduce noise from the data, but instead it applies ICA to reduce the noise from the vector loading matrix results obtained from the PCA.

The underlying assumption of this method is that not all variables in a biological system will contribute significantly to the biological process mechanisms, and therefore informative variables should be given priority over irrelevant variables in the loading matrix. Using IPCA to remove noise from the loading matrix is expected to produce a super-Gaussian data distribution based on the reduction of the non-Gaussian states of the PCA loading matrix to eliminate noise in the matrix by selecting the more informative components [13]. The IPCA algorithm introduced in uses the following steps [13]:

1. Implement singular value decomposition on a centered matrix *X*_(*n*×*p*)_ to obtain the loading matrix.
2. Select *m* components to reduce the dimensionality of the data from the PCA loading matrix results.
3. Implement fast ICA on the loading matrix to obtain an independent loading vector.
4. Take the projection *X*_(*n*×*p*)_ onto the *m* independent loading vectors to obtain IPCs.
5. Sort the PCs on the basis of the kurtosis value corresponding to the independent loading vector.

### Rotation forest ensemble classifiers

Due to their improved problem-solving abilities, MCSs have become a focus of both theoretical and practical attention in recent years, and MCSs are being increasingly used to optimize information retrieval from big data [11].

Bagging and Random Forest are MCS approaches that employ ensembles or combinations of decision trees. Bagging has two main stages: bootstrapping and aggregation. Bootstrapping is used for the random sampling of preliminary data used to build a compound tree. Aggregation is used to combine the estimation results obtained by bagging and merging all the alleged values into an alleged end value to represent the solution of a problem. Although bagging has been shown to be capable of reducing the predictive error rate for a single decision tree [21], it has disadvantages in cases in which the initial data correlation is very large, or the initial data have a high degree of noise. In such circumstances, bagging tends to produce a large variety of allegations, which results in inconsistency in making decisions [22]. To address this problem, in 2001, Breiman [21] proposed a new method, Random Forest, to improve the bagging method.

The fundamental difference between Random Forest and bagging lies in how the respective algorithms form combinations of decision trees. The free modifier used to perform separation on decision tree nodes is only one of an overall set of initial free variables produced as a result of random selection. The Random Forest process aims to produce decision trees of different sizes and shapes that should have reduced inter-tree correlations and, therefore, a smaller set of assumptions than under bagging [23].

In 2006, Rodriguez et al. [11] proposed a new ensemble classifier method known as Rotation Forest to simultaneously improve the accuracy and diversity of each classifier in the ensemble system. This method represents a modification of bagging and Random Forest methods based on the application of PCA to construct a rotational matrix that transforms initial variables into new variables to be used in constructing independent decision trees. Furthermore, the use of PCA ensures the diversity of the classifiers produced using this method [11], and all the major components resulting from the PCA process are retained to maintain the completeness of the information contained in the data [14].

In order to better understand how Rotation Forest works, we examined its application on a dataset *X* containing *n* observations and *p* features. We let *y*=[*y*_1_,*y*_2_,…,*y*_*n*_]^*T*^, where *y*_*j*_ represents the value of the class label set {*w*_1_,*w*_2_}. The decision tree in the ensemble is denoted by *D*_1_,…,*D*_*L*_ and the feature set of *X* is denoted by *F*. The two parameters applied—the number of decision trees used (L) and the number of feature subsets (K)—play a central role in determining the success of the Rotation Forest method.

The first step is to choose the number of decision trees (L) to be used. To establish the training data to build the decision tree *D*_*i*_,*i*=1,2,…,*L*, the following steps are taken:

1. Split *F* into *K* disjointed subsets at random, where *K* is a factor used to determine the value of *n* used to set the number of features contained in the feature subset as $M = \frac {n}{K}$.
2. Select the feature corresponding to a subset of *F*_(*i*,*j*)_ contained in the corresponding column from the training data *E*_*i*_ and then randomly select each nonempty subset to obtain a bootstrap object sample of 75% of the data.
3. Apply the PCA technique to use up to *M* features and *X* subsets from the selected *F*_(*i*,*j*)_ to order the coefficients of the PCs by size *M*×1 as $a_{(i,j)}^{(1)},a_{(i,j)}^{(2)},\dots,a_{(i,j)}^{(M)}$. Note that, because it is possible to generate some zero eigenvalues, fewer than *M* vectors can be obtained. PCA is performed across the sets to avoid duplication of the coefficients if the same feature subset is selected for different groups.
4. Construct a sparse rotation matrix *R*_*i*_ using the obtained coefficients as in Eq. 7.
5. Sort the columns of *R*_*i*_ according to the original feature sequence into a rearranged rotation matrix $R_{i}^{a}$. The transformed training set for classifier *E*_*i*_ is then given by $XR_{i}^{a}$.
6. Use $XR_{i}^{a}$ to build the set of classification trees *D*_*i*_.

7 $$ R_{i} = \left[\begin{array}{llll} a_{i,1}^{(1)},...,a_{i,1}^{(M1)} & 0 &... & 0\\ 0 & a_{i,2}^{(1)},...,a_{i,2}^{(M2)} &... & 0\\ \vdots & \vdots & \ddots & \vdots\\ 0 & 0 &... & a_{i,K}^{(1)},...,a_{i,K}^{(MK)}\\ \end{array}\right]  $$

The Rotation Forest (PCA) method developed in this study uses an algorithm corresponding to the original Rotation Forest algorithm proposed in [11], as described above, and it differs from the proposed Rotation Forest (IPCA) method only in terms of how the third step of the above algorithm is carried out. Following the basic IPCA approach described earlier in this paper, Rotation Forest (IPCA) performs IPCA analysis on *X*^∗^_*ij*_ and uses all the coefficients of the PCA loading matrix as inputs into the ICA method to obtain an independent loading vector (*S*^*T*^).

### Evaluation measures

To measure the performance of the proposed method, we applied five-fold cross-validation and several metrics—overall prediction accuracy, sensitivity, specificity, precision, and F1-score—which are defined as follows:

8 $$ Accuracy = \frac{TP+TN}{TP+FP+TN+FN},\\  $$

9 $$ Sensitivity = \frac{TP}{TP+FN},\\  $$

10 $$ Precision = \frac{TP}{TP+FP},\\  $$

11 $$ Specificity = \frac{TN}{TN+FP},\\  $$

12 $$ F1-score = 2 \times \frac{Sensitivity \times Precision}{Sensitivity + Precision},\\  $$

where true positive (TP) denotes the number of correctly predicted true PPIs between HIV-1 and human proteins, false negative (FN) denotes the number of true PPIs between HIV-1 and human proteins that were predicted to be noninteracting pairs, false positive (FP) denotes the number of true noninteracting pairs predicted to be PPIs, and true negative (TN) denotes the number of correctly predicted true noninteracting pairs.

## Abbreviations

DNA: Deoxyribonucleic acid
HIV-1: Human immunodeficiency virus type 1
ICA: Independent component analysis
IPCA: Independent principal component analysis
MCS: Multiple classifier systems
mRNA: Messenger ribonucleic acid
MS-PCI: Mass spectrometric protein complex identification
PC: Principal components
PCA: Principal component analysis
PPI: Protein-protein interaction
PseudoSMR: Pseudo-substitution matrix representation
RNA: Ribonucleic acid
SMR: Substitution matrix representation
Y2H: Yeast two-hybrid
